# A tool to predict survival in stage IV entero-pancreatic NEN

**DOI:** 10.1007/s40618-020-01404-4

**Published:** 2020-09-06

**Authors:** M. Tarquini, M. R. Ambrosio, M. Albertelli, P. B. de Souza, R. Gafà, I. Gagliardi, A. Carnevale, P. Franceschetti, M. C. Zatelli

**Affiliations:** 1 Department of Medical Sciences, Section of Endocrinology and Internal Medicine, University of Ferrara, Via Ariosto 35, 44100 Ferrara, Italy; 2 Endocrine Unit, Azienda Ospedaliero-Universitaria di Ferrara, Via Aldo Moro 8, Cona, 44124 Ferrara, Italy; 3 Endocrinology, Department of Internal Medicine DiMI, University of Genova, Genoa, Italy; 4 Pathology Unit, Department of Medical Sciences, University of Ferrara, Ferrara, Italy; 5 Department of Morphology, Surgery and Experimental Medicine, University of Ferrara, Ferrara, Italy

**Keywords:** Neuroendocrine neoplasms, NEP-Score, NEP-D, NEP-T, Survival

## Abstract

**Purpose:**

Well-differentiated stage IV neuroendocrine neoplasms (NEN) have an extremely heterogeneous, unpredictable clinical behavior. Survival prognostic markers, such as the recently proposed NEP-Score, would be very useful for better defining therapeutic strategies. We aim to verify NEP-Score applicability in an independent cohort of stage IV well-differentiated (WD) gastroentero-pancreatic (GEP) NEN, and identify a derivate prognostic marker taking into account clinical and pathological characteristics at diagnosis.

**Methods:**

Age, site of primary tumor, primary tumor surgery, symptoms, Ki67, timing of metastases of 27 patients (10 females; mean age at diagnosis 60.2 ± 2.9 years) with stage IV WD GEP NEN were evaluated to calculate the NEP-Score at the end of follow-up (NEP-T). We calculated the NEP-Score at diagnosis (NEP-D), which does not consider the appearance of new metastases during follow-up. Patients were subdivided according to whether they were alive or not at the end of follow-up (EOF) and an NEP-Score threshold was investigated to predict survival.

**Results:**

Mean NEP-T and mean NEP-D were significantly lower in 15 live patients as compared to 12 deceased patients (*p* < 0.01) at EOF. We identified an NEP-D = 116 as the cutoff that significantly predicts survival. No gender differences were identified.

**Conclusions:**

In our series, we confirmed NEP-Score applicability. In addition, we propose NEP-D as a simple, quick and cheap prognostic score that can help clinicians in decision making. NEP-D threshold can predict NEN aggressiveness and may be used to define the best personalized therapeutic strategy.

**Electronic supplementary material:**

The online version of this article (10.1007/s40618-020-01404-4) contains supplementary material, which is available to authorized users.

## Introduction

Epidemiological studies indicate that neuroendocrine neoplasms (NEN) are rare diseases, with a low incidence that increased over time in the last years [1–4]. Thanks to the improved therapeutic possibilities for NEN patients, prevalence is also increasing, being associated with different clinical outcomes according to grade, stage, age at diagnosis, and primary site of the tumor [1, 4–12]. Therapeutic management of gastroentero-pancreatic (GEP) NEN is mainly based on tumor differentiation and grading [5–7, 13–15], but markers of clinical outcome that could predict patient survival are still lacking [16]. Despite improvements in prognostic grading and staging systems, the challenge to predict outcome for patients with GEP NEN is difficult, particularly in stage IV GEP NEN, since these patients are often metastatic at the time of diagnosis and may display a wide spectrum of clinical behavior, ranging from indolent to aggressive, even within apparently homogeneous categories. Thus, selecting the optimal treatment is a challenging task. Blood-based biomarkers, such as chromogranin A (CgA) [8, 9, 12, 17, 18], circulating tumor cells and microRNAs [19], as well as tissue markers [20, 21] have been considered, but these putative markers have not been fully validated, so far. Pusceddu and co-workers [22] developed a classification prognostic score for overall survival (OS) in patients with stage IV well-differentiated (WD) G1–G2 NEN, named NEP-Score (NEuroendocrine Prognostic Score) by evaluating a monocentric cohort of patients with a long follow-up as training set (providing data reliability and homogeneity) and then two independent validation cohorts [8]. By using this score, they identified three different groups in terms of survival at 10 years: patients at low risk (NEP-Score ≤ 70) displayed > 70% survival; patients at intermediate risk (70 < NEP-Score ≤ 198) displayed 30–70% survival; patients at high risk (NEP-Score ≥ 199) displayed < 30% survival. In their study, NEP-Score turned out to be useful to rank patients according to their risk of death, predicting OS. The aim of the present study is to verify the applicability of the NEP-Score to an independent cohort of stage IV WD entero-pancreatic NEN, and to identify a derivate marker capable of predicting patients prognosis by taking into account clinical and pathological characteristics at diagnosis.

## Methods

### Study design

In this observational study, we retrospectively evaluated NEP-Score in a series of entero-pancreatic NEN patients referring to our center from 2008 to 2018. In line with previous studies [22], exclusively stage IV WD entero-pancreatic NEN patients with a follow-up > 24 months were included, while, as previously reported, patients with poorly differentiated neuroendocrine carcinomas (NEC G3) were excluded.

### Patients

We selected a group of 27 patients (10 females, 17 males; mean age at the diagnosis 60.2 ± 2.9 years; median age at diagnosis = 64 years; range 26–84 years) (Table 2) with stage IV WD entero-pancreatic NEN, characterized according to the 2019 WHO classification of tumors of the digestive system. Among these patients, 55% patients had documented progressive disease at the time of score calculation at diagnosis. All of them had been treated with long-acting somatostatin analogs and two patients with insulinoma had been treated with everolimus. Patients were evaluated for the following characteristics to calculate the NEP-Score at the end of follow-up (NEP-T) which lasted 70.3 ± 11.6 months: age, site of primary tumor, primary tumor surgery, symptoms, grading, timing of metastases, assigning the respective scores (see Table 1). A modified NEP-Score was then calculated, considered as the NEP-Score at diagnosis (NEP-D), which does not take into account the appearance of new metastases during follow-up. Patients were subdivided according to whether they were alive or not at the end of follow-up (EOF). Patient characteristics are displayed in Table 2 and in Supplemetary Table 1. This study is in accordance with the principles set out in the Declaration of Helsinki, has been specifically approved by the Local Ethics Committee (Comitato Etico Indipendente di Area Vasta Emilia Centro, CE-AVEC, at the Policlinico S.Orsola-Malpighi in Bologna) and authorized by the General Director of the Azienda Ospedaliero Universitaria in Ferrara (protocol number CE-AVEC 238/2020/Oss/AOUFe). Written consent was obtained from each patient or subject after full explanation of the purpose and nature of all procedures used.

**Table 1:** NEP-Score calculation (modified from Reference [22])

	Score
Age	
< 45	0
46–65	28
> 65	58
Site of primary tumor	
Ileum	0
Pancreas	59
Primary tumor surgery	
Yes	0
No	100
Functional status	
Yes	32
No	0
Ki67	
0–2	0
3–20	12
> 20	57
Timing of metastases	
Synchronous	0
Metachronous > 24 months	38
Metachronous ≤ 24 months	72

**Table 2:** Patients’ general features

	number	%
Gender		
Males	17	63
Females	10	37
Primary tumor site		
Ileum	13	48
Pancreatic	14	52
Metastasis timing		
Synchronous	12	44
Metachronous	15	56
Ki67 (Mib-1)		
0–2	17	63
3–20	7	26
> 20	3	11
Functional status		
No	18	67
Yes	9	33
Primary tumor surgery		
No	6	22
Yes	21	78
Alive at EOF		
No	12	44
Yes	15	56

### Statistical evaluation

Categorical data were summarized using frequencies and percentages. The Chi-square (χ^2^) test was performed to evaluate the presence of statistically significant differences among the evaluated groups in terms of NEP-Score. The paired Student’s *t* test was employed to compare the mean NEP-D and NEP-T scores among groups. A *p* value < 0.05 was considered significant. Sensitivity, specificity, positive predictive value (PPV), negative predictive value (NPV), and accuracy were calculated for each identified NEP-D and NEP-T threshold. All statistical analyses were performed using GraphPad software.

## Results

### NEP-T score calculation

**Fig. 1:**
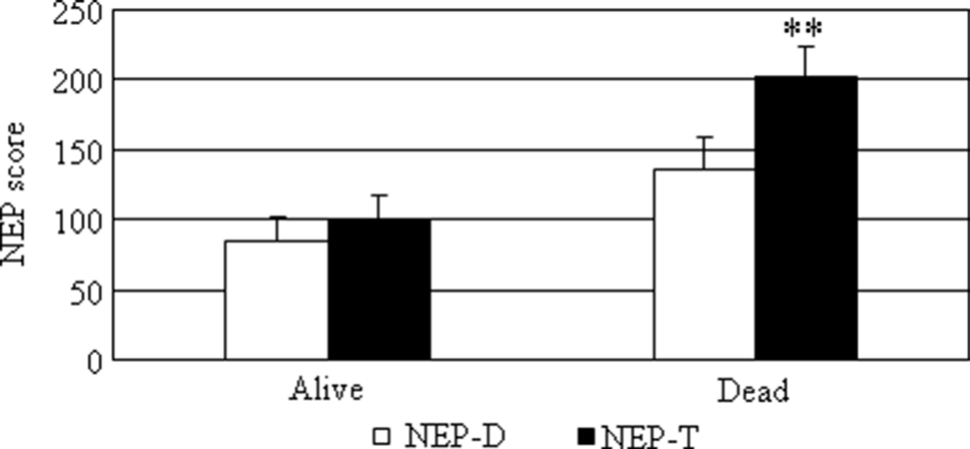
NEP-T and NEP-D scores. NEP-T (black columns) and NEP-D (white columns) scores are expressed as mean ± standard error of the mean (SEM). **p < 0.01 dead vs. alive patients at the end of follow-up

In our series, we found a mean NEP-T, corresponding to the original NEP-Score, of 145.5 ± 16.5. Patients were then subdivided according to whether they were alive or not at the EOF. We found a significant difference between mean NEP-T in live patients (100.3 ± 17.1; 15 patients) as compared to deceased patients at EOF (202.1 ± 21.3; 12 patients; *p* < 0.01) (Fig. 1).

### NEP-D score calculation

A modified NEP-Score was then calculated, considered as the NEP-Score at diagnosis (NEP-D), which does not take into account the appearance of new metastases during follow-up. In our series, we found a mean NEP-D of 108.0 ± 14.0. Patients were subdivided according to whether they were alive or not at EOF. We found that mean NEP-D in live patients (85.6 ± 16.7) was lower as compared to deceased patients (136.1 ± 22.5) at EOF, but this difference did not reach statistical significance (Fig. 1).

### Gender

We also considered a possible difference between genders. In male patients, mean NEP-T of the eight patients (mean age = 70.6 ± 2.8 years) who were alive at EOF was 69.5 ± 14.8, while the mean NEP-T of the nine patients (mean age = 64.3 ± 3.0 years) who were dead at EOF was significantly higher (203.3 ± 26.7; *p* < 0.01). This difference was not correlated to aging, since mean age at last follow-up in the two groups was not significantly different. Concerning female patients, mean NEP-T of the seven patients (mean age = 60.1 ± 2.6 years) who were alive at EOF was 135.4 ± 30.2, which was similar (198.3 ± 37.6; *p* = not significant) to the mean NEP-T of the three patients (mean age = 73.3 ± 1.7 years) who were dead at EOF. Similarly to males, there was no significant difference between the mean age of the two groups. Comparing males and females, we found that NEP-T in live patients was significantly lower in males as compared to females (*p* < 0.05). In addition, at EOF, 30% of female patients (3 out of 10) vs. 52.3% of male patients (8 out of 17) were dead. The difference in the proportion of live patients according to gender did not reach statistical significance. On the other hand, NEP-T in dead patients was similar in males and females, in keeping with a similar overall survival (OS = 51.6 ± 18.9 vs. 35.5 ± 10.8 months; *p* = not significant).

Concerning NEP-D, in male patients, mean NEP-D of live patients (mean age = 62.8 ± 4.7 years) was 55.8 ± 7.0, while the mean NEP-D of the patients who were dead at EOF (mean age = 60.0 ± 3.9 years) was significantly higher (139.3 ± 28.4; *p* < 0.01). Again, this difference was not correlated to age, since mean age at diagnosis in the two groups was not significantly different. Concerning female patients, mean NEP-D of live patients (mean age = 53.3 ± 8.8 years) at EOF was 119.7 ± 29.5, being similar to the mean NEP-D of patients (mean age = 70.3 ± 5.4 years) who were dead at EOF (126.3 ± 37.6; *p* = not significant). Similarly to males, there was no significant difference between the mean age of the two groups. Comparing males and females, we found that NEP-D in live patients was significantly lower in males as compared to females (*p* < 0.05). On the other hand, NEP-D in dead patients was similar in males and females, as reported for NEP-T.

### NEP-T threshold

**Fig. 2:**
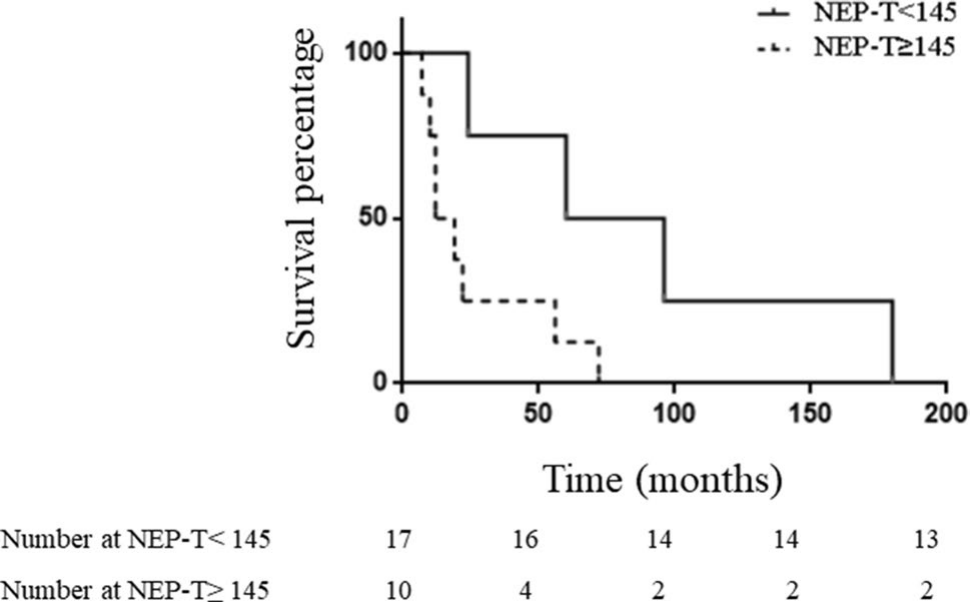
Patients’ survival according to the NEP-T threshold. Kaplan–Meier curves for survival of 27 patients with stage IV WD GEP NEN according to NEP-T threshold = 145

A NEP-T score threshold was investigated to assess the reliability of this score in detecting disease status. We found that a NEP-T score threshold ≥ 145 could correctly differentiate patients alive from those dead at EOF (Fig. 2). Indeed, 80% of patients with NEP-T ≥ 145 and 23.5% of patients with NEP-T < 145 were dead at EOF. This difference was statistically significant (*p* < 0.005). Similarly, OS was 3.5-fold shorter in patients with NEP-T ≥ 145 as compared to those with NEP-T < 145 (p < 0.05). A NEP-T ≥ 145 value represents a cutoff, allowing the best compromise between sensitivity (67%) and specificity (86.7%), with a PPV = 80%, an NPV = 76.5%, and accuracy = 77.8% to predict the outcome (dead vs. alive at EOF).

### NEP-D threshold

**Fig. 3:**
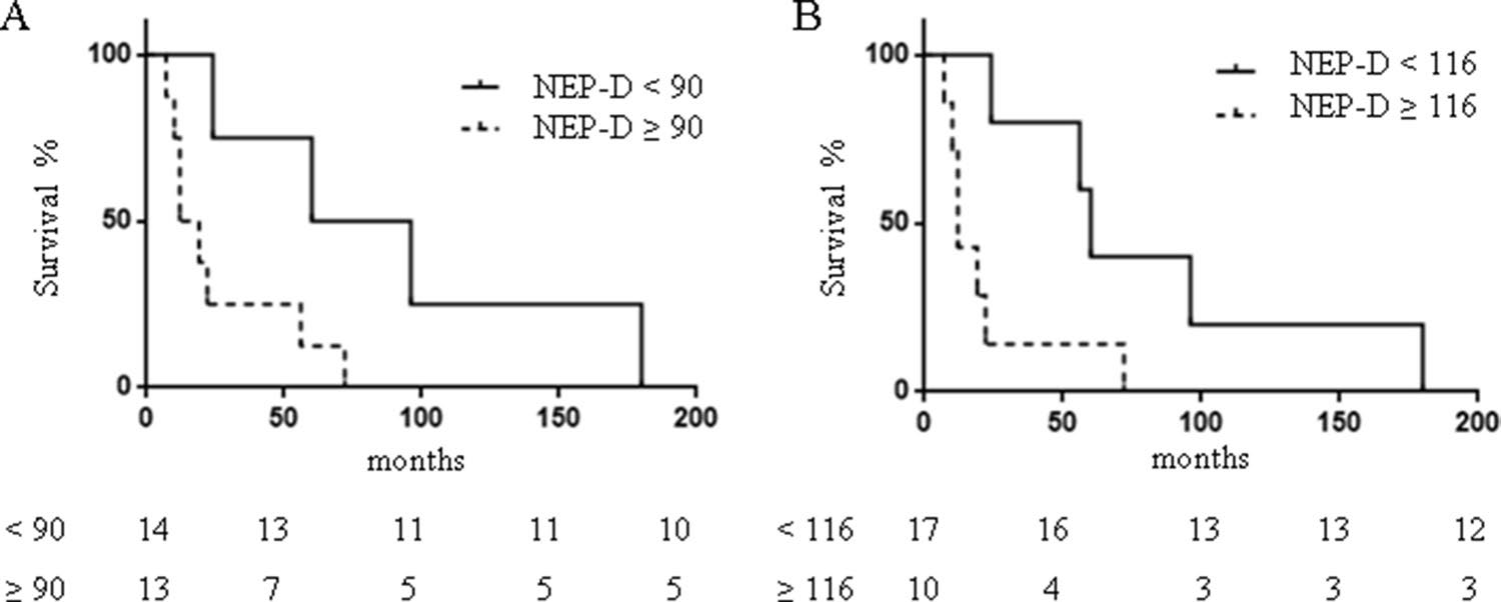
Patients’ survival according to different NEP-D thresholds. Kaplan–Meier curves for survival of 27 patients with stage IV WD GEP NEN according to the NEP-D threshold = 90 (**a**) and NEP-D threshold = 116 (**b**). **a** Survival curve of patients with NEP-D < 90 (continuous line) with OS = 90 ± 33 months or ≥ 90 (dotted line) with OS = 26.2 ± 8.5 months (*p* < 0.05). **b** Survival curve of patients with NEP-D < 116 (continuous line) with OS = 83.2 ± 26.8 months or ≥ 116 (dotted line) with OS = 22.0 ± 8.6 months (*p* < 0.05)

A NEP-D score threshold was investigated to predict survival. We found that a value of NEP-D ≥ 90 represents a cutoff, allowing the best compromise between sensitivity (67%) and specificity (67%), with a PPV = 61%, a NPV = 71%, and accuracy = 82% to predict the outcome (dead vs. alive at EOF). Indeed, at EOF, 61.5% of patients with NEP-D ≥ 90 and 28.5% of patients with NEP-D < 90 were dead. However, statistical evaluation did not show any significance. Mean OS of patients dead at EOF was 47.5 months; in patients with NEP-D < 90 mean OS was 90 ± 33 months, while in patients with NEP-D ≥ 90 mean OS was significantly shorter, corresponding to 26.2 ± 8.5 months (Fig. 3a; *p* < 0.05).

On the other hand, a value of NEP-D ≥ 116 displays a moderate sensitivity (58%) with a specificity of 80%, PPV = 70%, NPV = 71%, and accuracy = 70% to predict the outcome (dead vs. alive at the EOF). At EOF 70% of patients with NEP-D ≥ 116 and 29.5% of patients with NEP-D < 116 were dead (p < 0.05). In patients with NEP-D < 116 mean OS was 83.2 ± 26.8 months, while in patients with NEP-D ≥ 116 mean OS was significantly shorter (22.0 ± 8.6 months; *p* < 0.05) (Fig. 3b).

Moreover, we calculated the difference between NEP-T and NEP-D for each patient as a “Delta-NEP score”. In patients who were alive at EOF mean Delta-NEP score was 13 ± 7.2, while in patients who were dead at EOF mean Delta-NEP score was significantly higher (56 ± 8.2; *p* value < 0.05).

### Patients’ survival

During follow-up (70.3 ± 11.6 months), 12 patients died due to disease progression, with an overall mortality of 44.4% and a mean OS = 51.2 ± 14.9 month. We investigated whether the identified NEP-D thresholds may be useful to predict 5 years survival: we found that 5 out of 6 patients with NEP-D score < 90 were alive, while 4 out of 11 patients with NEP-D score ≥ 90 were alive 5 years after diagnosis. Similar results were found with the NEP-D threshold set at 116: 7 out of 12 patients with NEP-D score < 116 and 2 out of 9 patients with NEP-D score ≥ 116 were alive 5 years after diagnosis. However, the observed different distribution did not reach statistical significance in both cases, probably due to the low patient number.

When evaluating mean Delta-NEP score, we found that in patients with ileal NEN (38.7 ± 10.3), this score was not significantly different from that of patients with pancreatic NEN (pNEN) (36.2 ± 9.2). On the other hand, patients with ileal NEN showed a sixfold longer mean OS as compared to patients with pNEN (81.3 ± 21.9 vs. 13.6 ± 2.3 month; *p* < 0.05) in keeping with previous reports [16].

## Discussion

Biological behavior of GEP NEN is very heterogeneous and several factors associated with both the patient and the tumor may modify prognosis. The possibility of identifying a score that could predict GEP NEN patients’ prognosis is of great interest for clinicians. Despite that the majority of GEP NEN are characterized by an indolent clinical course, a subgroup displays an unpredictable behavior, characterized by an unfavorable outcome and worse survival as compared to patients with the same characteristics. Patients with WD stage IV disease may be treated by several approaches, including somatostatin analogs (SSA), chemotherapy, peptide receptor radionuclide therapy (PRRT), and targeted therapies, even though a sequence has not been precisely established so far [6, 15, 23–25]. However, these approaches may temporarily control disease progression, which frequently follows the first therapeutic line [6, 15]. Therefore, it would be very important to identify those patients that may benefit by an aggressive treatment from those who could be spared from unnecessary therapy. Consolidated prognostic factors are represented by morphology (i.e., differentiation), proliferation rate in terms of number of mitosis and proliferation index [5, 7, 10, 13, 14, 26, 27]. Currently, Ki67 is considered one of the most important prognostic factors in NEN [5, 7, 14, 26, 28]. However, useful criteria to subdivide stage IV GEP NEN are not available to precisely assess prognosis and metastatic burden value.

Classification systems are continuously updated, contributing to a better identification of prognostic characteristics of each NEN category. However, few studies have investigated prognostic indexes capable of estimating survival in stage IV GEP NEN. The prognostic NEP-Score identified by Pusceddu et al. was found to be useful to stratify survival probability also in very heterogeneous patients groups, but could be improved by including additional information, such as biochemical [8, 9, 11, 12, 17], molecular [5, 19, 29–32] and immunohistochemical markers [20, 21], imaging characteristics [33, 34], gender [8, 9, 35, 37], ethnicity [8, 9], nodal involvement, tumor size [8, 9, 11, 12, 28, 35], previous treatments [8, 9], and metastatic disease burden [8, 9, 11, 16]. In addition, the influence of medical treatment outcome and of therapeutic advances developed during the last years [23–25, 36] were not taken into account when considering OS.

In our study, we validated the NEP-Score in an independent homogeneous series of 27 patients with stage IV WD entero-pancreatic NEN followed up in our center from 1998 to 2018. We confirm the validity of the score, since our NEP-T, corresponding to the NEP-Score in the study by Pusceddu et al., was found to be higher in patients dead as compared to patients alive at EOF. This finding confirms that NEP-T score can be adopted in very different clinical settings. However, its utility is impaired by the fact that a 24 months follow-up is needed to calculate the score. We therefore modified the score, taking into account only the characteristics available at diagnosis, obtaining the NEP-D score that does not consider the development of metachronous metastases. We then investigated whether NEP-D may predict survival, also evaluating gender differences. We found that NEP-D is significantly higher in male patients that are dead as compared to those alive at EOF, independently of age. This finding was not replicated in females, probably due to the low subject number in the group of patients dead at EOF (only three patients). In the group of patients alive at EOF, males displayed a significantly lower mean NEP-D as compared to females, probably due to a greater prevalence in the latter group of pNEN, a primary site associated with a greater score as compared to ileal NEN. In the group of patients dead at EOF, no significant differences were found between genders in terms of NEP-D. These results indicate that NEP-D does not depend on patients’ gender, suggesting that stage IV NEN entero-pancreatic patients prognosis is not influenced by gender. This hypothesis is further strengthened by the evidence that OS is similar in both genders. Our data are not in line with previous evidence reporting a protective role for female gender in patients aged > 75 years with pNEN [37]. This difference may be due to patient selection in our study, which includes subjects with a wider age range (26–84 years) with only five patients > 75 years (2 F and 3 M). Therefore, these studies cannot be compared.

The prognostic significance of metastatic disease burden and progression at metastatic sites is difficult to assess. The study by Pusceddu et al. allows to quantify this item by assigning a score that we validated in our cohort. We then developed a score that does not depend on this item, and set out to identify a threshold that could predict survival since the diagnosis. The identified thresholds have slightly different performance, with an NEP-D of 90 representing the best compromise between sensitivity and specificity. However, this threshold did not reach statistical significance, probably due to the low number of investigated patients. This does not hold true for the NEP-D threshold of 116 that, however, displays a worse sensitivity. Therefore, this threshold may underestimate NEP-D value as a marker of survival. On the other hand, they may be useful to predict 5 years survival at the time of diagnosis, providing further help to plan a patient-tailored management. However, these thresholds need to be validated in independent cohorts of patients with stage IV entero-pancreatic NEN.

Delta-NEP score may offer more indications, since it measures the development of new metastases, with a different score depending on the timing (before or after 24 months from diagnosis). The evidence that this score is significantly higher in patients dead as compared to those alive at EOF suggests that metastases development in new sites profoundly influences survival in stage IV NEN GEP patients, being more important than the progression of metastases already present at diagnosis. The evidence that ileal and pNEN do not differ concerning this parameter suggests that metastatic disease progression was similar in the two groups and confirms the pancreatic site as an independent unfavorable prognostic marker. This hypothesis is further strengthened by the evidence that patients with ileal NEN showed a sixfold longer mean OS as compared to patients’ pNEN, in keeping with previous reports [16].

In conclusion, our study suggests NEP-D as a simple, quick, cheap, easy to calculate prognostic score that employs information readily available and, at the same time, provides help to clinicians in decision making. This score encloses in a single index the characteristics known to influence prognosis in GEP NEN (grade, stage, age at diagnosis, primary site) [7–12, 16, 26, 27].

NEP-D application could predict the aggressiveness of each NEN to better identify those patients that will benefit from an aggressive and timely treatment as well as those with an indolent disease that could be spared from overtreatment.

## Electronic supplementary material

Below is the link to the electronic supplementary material.

Supplementary file1 (DOC 70 kb)

## Abbreviations

WD: Well differentiated
OS: Overall survival
GEP NEN: Gastroentero-pancreatic neuroendocrine neoplasms
NEP-Score: Neuroendocrine prognostic score
EOF: End of follow-up
