# Supplementary material for: A tool to predict survival in stage IV entero-pancreatic NEN
Source: J Endocrinol Invest. 2020 Sep 6;44(6):1185–92. doi: 10.1007/s40618-020-01404-4 (PMC8124053; doi:10.1007/s40618-020-01404-4)
Supplement: Supplementary file 1 — Supplementary file1 (DOC 70 kb) [file 40618_2020_1404_MOESM1_ESM.doc]

Supplementary table 1: Patients characteristics.

| n° | gender | Site of primary tumor | Age at diagnosis | Grading | Syndrome | Primary tumor surgery | Timing of metastasis | NEP-D | NEP-T | Dead |
| --- | --- | --- | --- | --- | --- | --- | --- | --- | --- | --- |
| 1 | F | Ileum | 64 | G1 | Yes | Yes | Metachronous | 60 | 132 | Yes |
| 2 | F | Pancreas | 66 | G2 | No | Yes | Metachronous | 129 | 201 | Yes |
| 3 | F | Ileum | 81 | G1 | Yes | No | Metachronous | 190 | 262 | Yes |
| 4 | F | Pancreas | 84 | G1 | No | No | Synchronous | 217 | 217 | No |
| 5 | F | Ileum | 42 | G1 | No | Yes | Synchronous | 0 | 0 | No |
| 6 | F | Pancreas | 54 | G2 | Yes | Yes | Synchronous | 131 | 131 | No |
| 7 | F | Pancreas | 31 | G2 | No | Yes | Metachronous | 71 | 143 | No |
| 8 | F | Pancreas | 72 | G1 | No | No | Synchronous | 217 | 217 | No |
| 9 | F | Pancreas | 26 | G2 | Yes | Yes | Synchronous | 103 | 103 | No |
| 10 | F | Pancreas | 64 | G2 | No | Yes | Metachronous | 99 | 137 | No |
| 11 | M | Pancreas | 66 | G1 | No | No | Synchronous | 217 | 217 | Yes |
| 12 | M | Ileum | 62 | G1 | Yes | Yes | Metachronous | 60 | 132 | Yes |
| 13 | M | Ileum | 55 | G1 | Yes | Yes | Metachronous | 60 | 132 | Yes |
| 14 | M | Ileum | 54 | G1 | Yes | Yes | Metachronous | 60 | 132 | Yes |
| 15 | M | Pancreas | 71 | G2 | No | Yes | Metachronous | 129 | 201 | Yes |
| 16 | M | Ileum | 70 | G1 | Yes | Yes | Metachronous | 90 | 162 | Yes |
| 17 | M | Pancreas | 75 | G3 | No | No | Metachronous | 274 | 346 | Yes |
| 18 | M | Pancreas | 45 | G3 | No | Yes | Metachronous | 116 | 188 | Yes |
| 19 | M | Pancreas | 42 | G3 | Yes | No | Metachronous | 248 | 320 | Yes |
| 20 | M | Ileum | 67 | G2 | No | Yes | Metachronous | 70 | 142 | No |
| 21 | M | Ileum | 58 | G1 | No | Yes | Synchronous | 28 | 28 | No |
| 22 | M | Ileum | 78 | G1 | No | Yes | Synchronous | 58 | 58 | No |
| 23 | M | Pancreas | 47 | G1 | No | Yes | Metachronous | 87 | 159 | No |
| 24 | M | Ileum | 68 | G1 | No | Yes | Synchronous | 58 | 58 | No |
| 25 | M | Pancreas | 41 | G1 | No | Yes | Synchronous | 59 | 59 | No |
| 26 | M | Ileum | 65 | G1 | No | Yes | Synchronous | 28 | 28 | No |
| 27 | M | Ileum | 78 | G1 | No | Yes | Synchronous | 58 | 58 | No |
